# Molecular Insight into the Pharmacological Potential of *Clerodendrum minahassae* Leaf Extract for Type-2 Diabetes Management Using the Network Pharmacology Approach

**DOI:** 10.3390/medicina59111899

**Published:** 2023-10-26

**Authors:** Fatimawali, Trina Ekawati Tallei, Billy Johnson Kepel, Widdhi Bodhi, Aaltje Ellen Manampiring, Firzan Nainu

**Affiliations:** 1 Department of Pharmacy, Faculty of Mathematics and Natural Sciences, Sam Ratulangi University, Manado 95115, North Sulawesi, Indonesia; 2 Department of Biology, Faculty of Mathematics and Natural Sciences, Sam Ratulangi University, Manado 95115, North Sulawesi, Indonesia; 3 Department of Biology, Faculty of Medicine, Sam Ratulangi University, Manado 95115, North Sulawesi, Indonesia; 4 Department of Chemistry, Faculty of Medicine, Sam Ratulangi University, Manado 95115, North Sulawesi, Indonesia; billy.kepel@unsrat.ac.id (B.J.K.); widdhibodhi@unsrat.ac.id (W.B.); aldamanampiring@unsrat.ac.id (A.E.M.); 5 Department of Pharmacy, Faculty of Pharmacy, Hasanuddin University, Makassar 90245, South Sulawesi, Indonesia; firzannainu@unhas.ac.id

**Keywords:** network pharmacology, *Clerodendrum minahassae*, type-2 diabetes, insulin signal transduction, PIK3R1

## Abstract

*Background and Objectives:* The increasing occurrence and prevalence of type-2 diabetes mellitus (T2DM) have led to a growing interest in researching available treatment alternatives. *Clerodendrum minahassae*, a native plant species of North Sulawesi, has been a focus of ethnopharmacological studies due to its significance contributions to drug development, particularly its potential antidiabetic properties. This study investigated the pharmacological potential of *Clerodendrum minahassae* (CM) leaf extract for managing type-2 diabetes (T2DM) using a network pharmacology approach. *Materials and Methods*: Active compounds were extracted from CM leaves, and their interactions with target proteins in T2DM were explored through various in silico analyses. *Results*: SAR analysis using Way2Drug Pass Online identified 29 bioactive CM leaf extract compounds with promise as T2DM treatments. Additionally, 26 of these met Ro5 criteria for favorable drug-likeness. Most compounds exhibited positive pharmacodynamic and pharmacokinetic profiles, with 22 considered safe, while 7 posed potential toxicity risks when ingested individually. CM leaf extract targeted 60 T2DM-related proteins, potentially affecting T2DM via cytokine regulation, particularly in proteins linked to metabolic processes, cellular response to angiotensin, and the sphingosine-1-phosphate signaling pathway. The network pharmacology analysis identified five genes targeted by CM leaf extract, namely, STAT3, MAPK1, ESR1, PIK3R1, and NFKB1. Among these genes, PIK3R1’s interaction with the insulin receptor (INSR) positions it as a crucial candidate gene due to its pivotal role in insulin signal transduction during T2DM development. *Conclusions*: This research sheds light on the therapeutic potential of CM leaf extract for treating T2DM. This potential is attributed to the diverse array of bioactive compounds present in the extract, which have the capacity to interact with and inhibit proteins participating in the insulin signal transduction pathway crucial for the progression of T2DM. The findings of this study may open up possibilities for future applications of CM leaf extract in the development of novel T2DM treatments.

## 1. Introduction

In 2021, the global population living with diabetes was estimated at 529 million, with a confidence interval between 500 million and 564 million. The worldwide age-standardized total prevalence of diabetes was 6.1%, with a range between 5.8% and 6.5% [1]. The prevalence of diabetes is anticipated to persistently increase, as evidenced by projections indicating that around 642 million individuals are anticipated to be affected by diabetes by the year 2040 [2]. The prevalence of diabetes mellitus (DM) among adults aged 20 years in Asia and Australia (Western Pacific) has reached a total of 162.6 million cases, accounting for approximately 9.6% of the overall population in Southeast Asia. This alarming figure is accompanied by a significant number of deaths, with 1.2 million individuals succumbing to the disease. According to recent data, Indonesia is positioned as the sixth country with the highest number of DM cases among those aged 15 years and older. The prevalence of this condition in Indonesia is reported to be 10.9% [3]. There are several elements that are acknowledged to have a role in the onset of diabetes and its associated complications. Additionally, there is a growing recognition of the involvement of the immune system in the development of metabolic illnesses. In the context of type-1 diabetes mellitus (T1DM), it has been widely recognised that inflammation is a key factor in the pathophysiology of pancreatic islets. However, recent findings suggest that inflammation also has a significant impact on the development of type-2 diabetes mellitus (T2DM) [4,5].

The treatment of diabetes with synthetic drugs often leads to adverse effects include gastrointestinal reactions, weight gain, fluctuations in blood glucose levels [6,7], and an increased risk of cardiovascular diseases [8,9]. Furthermore, synthetic drugs typically fail to effectively address serious biochemical disturbances and diabetes-related complications. In recent times, several novel synthetic antidiabetic drugs have been developed and introduced to treat hyperglycemic conditions in diabetic patients. However, many of these drugs carry the potential for adverse effects [10]. As a result, the long-term treatment of T2DM using synthetic drugs is limited. In contrast, traditional herbal remedies for diabetes are used worldwide and offer a promising alternative for managing T2DM [11]. Due to their lower cost and greater accessibility compared to synthetic drugs, herbal therapies have been extensively utilized in the treatment of T2DM [12]. More than 1200 species of medicinal plants are employed globally in traditional medicine for their antidiabetic properties [13]. These therapeutic options can be utilized as antidiabetic agents in conjunction with other antidiabetic drugs or insulin, with minimal side effects [14].

Compared to synthetic drugs, natural products play a significant role in the treatment of DM. The therapeutic benefits of natural products are attributed to their multi-component and multi-target actions, which result in combined or synergistic effects. With the rapid advancement of new technologies, there has been an increasing focus on research related to natural antidiabetic products. The approach of using multi-target drugs holds a promising future, especially when monotherapy appears to be ineffective. The concept of poly-pharmacology has also been well-received and embraced by pharmaceutical companies and researchers, with a focus on the development of multi-target drugs [15].

Advancements in bioinformatics, network pharmacology, and the integration of biological and pharmacological networks are considered promising approaches towards the development of cost-effective drugs that can uncover the underlying relationships between multi-components and multi-targets. Network pharmacology approaches have been developed to identify effective drugs for the treatment of T2DM and related metabolic disorders, such as obesity and metabolic syndrome [16].

Most plants contain bioactive compounds, including carotenoids, flavonoids, terpenoids, alkaloids, and glycosides [17,18,19,20], and they often exhibit antidiabetic effects [21]. *Clerodendrum minahassae* (Teisjm. and Binn.), abbreviated as CM, is a plant species native to the region of North Sulawesi [22]. Ethno-pharmacological approaches have revealed that the genus *Clerodendrum* plays various significant roles in drug development, including anti-inflammatory, antidiabetic, antibacterial, and antioxidant properties [23,24]. Several studies have reported the presence of phytochemical compounds, such as alkaloids, flavonoids, steroids, saponins, phenols, and tannins, in the ethanolic extract of CM leaves [25,26].

The objective of this research was to analyze the bioactive compounds of the CM leaf extract using network pharmacology to gain a molecular understanding of its pharmacological potential for managing T2DM. Mechanisms related to T2DM, including the inhibition of amylase and glucosidase, targeting of beta-cell dysfunction, the AMPK signaling pathway, and the PI3K/Akt signaling pathway, are summarized and discussed. This research highlights the potential of the network pharmacology approach as it not only provides a novel research paradigm for natural products but also enhances current strategies for discovering antidiabetic drugs. Furthermore, it emphasizes the perspective of possible applications of network pharmacology in diabetes therapy and related drug discovery.

## 2. Materials and Methods

### 2.1. Bioactive Compound Analysis of CM Leaves

The CM leaves were obtained from the South Minahasa garden, located at an altitude of 210 m above sea level. The specimen was authenticated by a botanist at the Department of Biology, Faculty of Mathematics and Natural Sciences, Sam Ratulangi University. The collected leaves were then subjected to a drying process to obtain dried simplicia, which refers to the raw medicinal materials in their dried, whole, or unprocessed state. Subsequently, the dried simplicia underwent fine powdering. To extract the bioactive compounds from CM leaves, two types of solvents were employed: a polar solvent, specifically 96% p.a. ethanol, and a non-polar solvent, n-hexane. The analysis of the bioactive compounds was conducted using the gas chromatography–mass spectrometry (GC-MS) (TRACE 1310 GC coupled with ISQ LT Single Quadrupuole MS, Thermo Scientific^™^, Thermo Fisher Scientific Inc., Waltham, MA, USA), following the procedure outlined in our previous research [27]. The identification of the bioactive compounds present in CM leaves was based on the data acquired from the GC-MS analysis.

### 2.2. Profiling of CM Extract

The PubChem database (https://pubchem.ncbi.nlm.nih.gov/, accessed on 7 July 2023) was utilized to retrieve the Simplified Molecular-Input Line-Entry System (SMILES) profiles and three-dimensional (3D) structures of each component discovered in the GC-MS analysis of the CM leaf extract.

### 2.3. Prediction of Bioactive Compound Activities

The compounds present in the CM leaf extract were subjected to potency analysis using the WAY2DRUG PASS prediction tool (http://www.pharmaexpert.ru/passonline/predict.php, accessed on 8 July 2023) for antidiabetic treatment, specifically targeting T2DM. This tool employs SAR analysis to compare the input compounds with known compounds that exhibit specific potency. A higher degree of structural similarity between the input compound and known compounds results in a correspondingly higher prediction score. Compounds exhibiting similar structures can be predicted to possess similar potential bioactivities. The Pa (probability to be active) value represents the output prediction score obtained from the web, indicating the potential of the tested compound. A Pa value exceeding 0.7 suggests that the compound is predicted to have high potential as, for instance, an anti-inflammatory agent, owing to its resemblance to compounds in the database. It is recommended to utilize a cutoff score of 0.5 for assessment. The Pa value reflects the accuracy of the obtained prediction function, where higher Pa values indicate greater accuracy [28,29].

### 2.4. Pharmacokinetic Analysis

ADMET (absorption, distribution, metabolism, excretion, and toxicity) represents a set of pharmacokinetic parameters crucial in drug development, assessing how a drug behaves in the body, including absorption into the bloodstream, distribution to tissues, metabolism by enzymes, excretion, and potential toxic effects [30,31,32]. The drug-likeness characteristics were determined for each ligand based on the Lipinski’s Rule of 5 (Ro5), which was analyzed using the Protox II database [33] (https://tox-new.charite.de/protox_II/index.php?site=compound_input, accessed on 12 July 2023) and the ADMETLab 2.0 database [34,35] (https://admetmesh.scbdd.com/service/evaluation/index, accessed on 12 July 2023). The SMILES notation of each ligand served as the input for both databases.

### 2.5. Protein Target Identification and Analysis

Target analysis of the CM leaf extract was performed using the SEA target analysis tool (https://sea.bkslab.org/, accessed on 13 July 2023) and SuperPred (https://prediction.charite.de/, accessed on 13 July 2023). Target predictions were obtained by inputting the SMILES notation identified in the previous stages. The cut-off score for SEA Target was set at >0.4 (ranging from 0 to 1), while SuperPred’s cut-off scores for probability and model accuracy were both set at 80% (ranging from 0 to 100%). Genes and proteins associated with T2DM were retrieved from the Open Targets database (https://www.opentargets.org/, accessed on 14 July 2023). Disease-related targets and the targets from the CM leaf extract were then mapped using a Venn diagram to determine the intersection of targets. The annotation of targets from the CM leaf extract was carried out using the DAVID webserver (https://david.ncifcrf.gov/, accessed on 15 July 2023) with a focus on biological processes and Kyoto Encyclopedia of Genes and Genomes (KEGG) pathways.

### 2.6. Network Pharmacology Analysis

The interaction analysis between target proteins obtained from the CM leaf extract and their relation to T2DM was carried out utilizing the STRING (Search Tool for the Retrieval of Interacting Genes/Proteins) Database Version 11.5. The input consisted of the target proteins derived from the CM leaf extract along with the intersection of proteins associated with T2DM, including the insulin receptor (INSR). The organism chosen for analysis was *Homo sapiens* (human), and a high confidence score threshold of 0.7 was applied to ensure robust interactions. The resultant analysis data, presented in TSV format from the STRING database, were further processed using CytoScape Version 3.10 for in-depth investigation. CytoScape served as a valuable tool for network analysis, enabling the exploration of key network parameters such as degree, betweenness centrality, and closeness centrality.

## 3. Results

### 3.1. Profile of CM Leaf Extract

Table 1 presents the compound profile of CM leaf extract obtained through GC-MS analysis, encompassing 29 identified compounds (C1 to C29) classified based on the type of solvent utilized during analysis: non-polar (n-hexane) and polar (ethanol). The non-polar compounds (C9 to C16) consist of aliphatic hydrocarbons and derivatives, while the polar compounds (C17 to C29) comprise a diverse array of compounds, including phytol, fatty acid esters, and oxygenated compounds. Two compounds, hexadecanoic acid methyl ester (C7) and phytol (C26), were detected in both solvents, suggesting their dual hydrophobic and hydrophilic properties. The results imply the complexity of CM leaf extract’s chemical composition and its potential for diverse biological activities. The identified bioactive compounds, such as fatty acid esters, may align with traditional medicinal uses of CM.

### 3.2. Potential of CM Leaf Extract for T2DM Treatment

**Figure 1:**
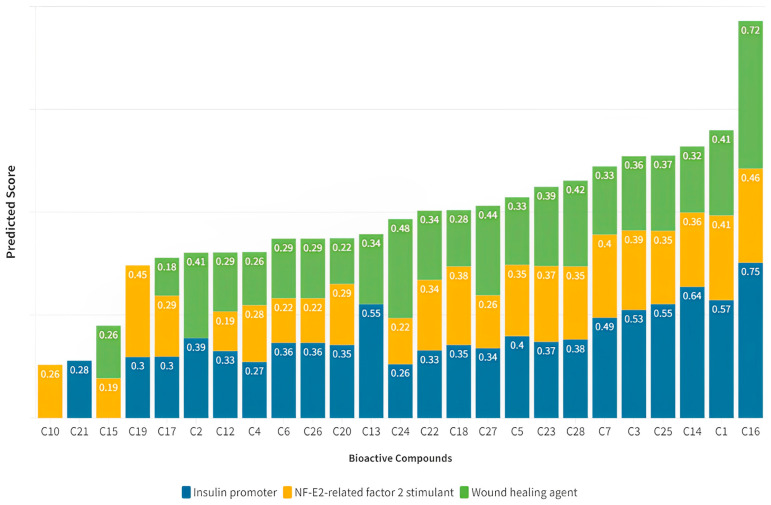
The evaluation of CM leaf extract’s potential for T2DM treatment based on structure–activity relationship (SAR) predictions. The assessment included its impact as an insulin promoter, Nrf2 stimulant, and wound healing agent. Notably, compound C16 (α-amyrin) demonstrated the most consistent effects.

The parameters used to assess the potential of CM leaf extract as a treatment for T2DM include its effects as an insulin promoter, nuclear-factor-erythroid-2-related factor 2 (Nrf2) stimulant, and wound healing agent. Compound C16 (α-amyrin), identified as a bioactive compound in the CM leaf extract, exhibited the most consistent values compared to other compounds (Figure 1). A research investigation was conducted to explore the correlation between Nrf2, inflammatory cytokines, and clinical remission in patients with T2DM [33]. Blood samples were obtained from a cohort of 180 T2DM patients (comprising 105 males and 75 females) and 150 control subjects (consisting of 86 males and 64 females). The study’s findings revealed that within the T2DM samples, there was a significant increase in the levels of Th1/Th2 cytokines and markers associated with oxidative stress, alongside a decrease in Nrf2 expression and its downstream targets within the peripheral blood mononuclear cells (PBMCs). Furthermore, the study demonstrated that the activation of Nrf2 led to the restoration of impaired insulin secretion in β-cells caused by cytokine-induced stress, as evaluated through glucose-stimulated insulin secretion (GSIS) assessment. A study reported that a high-fructose diet (HFD) in rats caused adverse metabolic changes, but α-amyrin administration at different doses mitigated these effects, showing a dose-dependent response. α-Amyrin also reduced hepatic oxidative stress and preserved peroxisome-proliferator-activated receptor alpha (PPAR-α) expression in the liver. The study indicates α-amyrin’s potential in attenuating metabolic syndrome induced by an HFD in rats [34].

**Figure 2:**
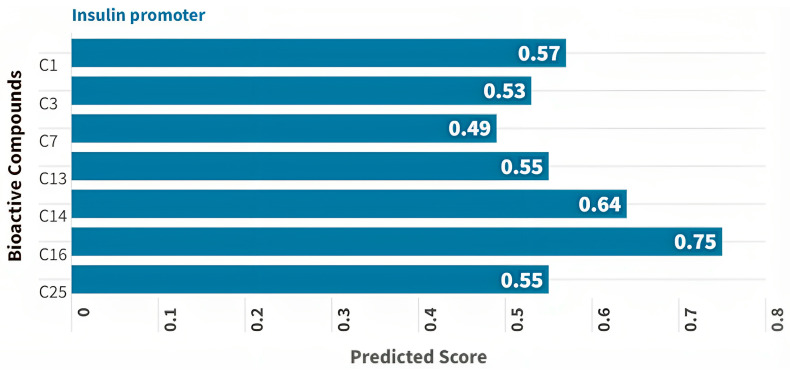
SAR-based prediction of CM leaf extract’s potential for treating T2DM, particularly as an insulin promoter. Seven compounds exhibited Pa values greater than 0.4. Remarkably, compound C16 stood out with the highest predicted value as an insulin promoter (Pa score of 0.75) and as an Nrf2 stimulant (Pa score of 0.46).

The prediction for T2DM treatment is primarily based on its potential as an insulin promoter. This is because almost all compounds exhibit a consistent insulin-promoting potential. Compounds with a predicted insulin-promoting value >0.4 were further subjected to network analysis. Among the compounds, seven were found to have Pa values > 0.4. Notably, compound C16 demonstrated the highest predicted value as an insulin promoter (Pa score of 0.75) and as an Nrf2 stimulant (Pa score of 0.46) (Figure 2). The seven compounds with a Pa score > 0.4 for insulin promotion also met the Ro5 criteria (Table 2).

### 3.3. Pharmacokinetic Evaluation and Drug-Likeness Assessment

In the drug discovery process, the assessment of ADMET plays a vital role in evaluating the potential of drug candidates. To obtain predictions for crucial pharmacokinetic parameters, the ADMETlab database was employed, which includes Ro5 and ADME/T predictions for a given compound. Additionally, the Protox II database [35] was employed to assess the compound’s compliance with Lipinski’s rule and its potential toxicity. Lipinski’s Ro5 results, as shown in Table 3, facilitate the differentiation of molecules as drug-like or non-drug-like based on specific properties.

**Figure 3:**
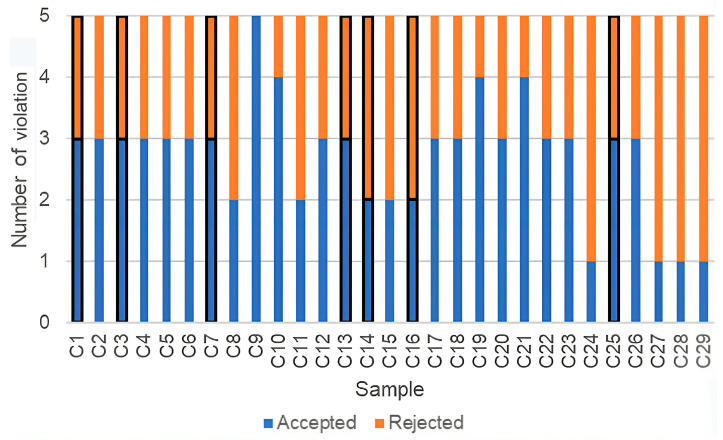
Prediction of compound drug-likeness within the CM leaf extract using Lipinski’s Rule of Five (Ro5). The bars outlined in black indicate the seven compounds selected for additional analysis. Out of the 29 bioactive compounds assessed, 26 were found to conform to the Ro5 criteria, indicating their potential as drug-like compounds.

Among the 29 bioactive compounds analyzed in the sample, 26 demonstrated adherence to the Ro5 rule, suggesting their potential drug-likeness, as depicted in Figure 3. However, it is essential to recognize that the absence of drug-likeness in the remaining five compounds does not necessarily preclude their potential as drugs. Instead, these compounds might necessitate higher energy requirements or rely on mechanisms involving active transport to achieve intracellular localization. Furthermore, the ADMET analysis indicated that nearly all compounds exhibited favorable pharmacodynamics and pharmacokinetics properties.

**Figure 4:**
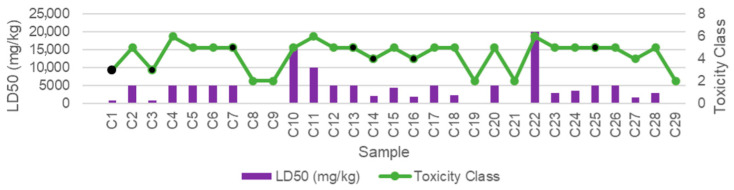
Toxicity prediction based on LD_50_ and toxicity classes of each compound detected in CM leaf extract. The black-filled labels indicate the seven compounds that are the focus of further analysis. Among the 22 bioactive compounds, the average LD_50_ and toxicity classes spanned from Class IV to Class VI, indicating that these compounds are predicted to have a low toxicity level and are considered safe.

The toxicity of compounds can be categorized into six classes [35] based on their lethal dose (LD_50_) when swallowed. Class I includes compounds that are fatal if swallowed with LD_50_ ≤ 5, while Class II consists of compounds with LD_50_ ranging from 5 to 50, also fatal if swallowed. Class III includes compounds with LD_50_ ranging from 50 to 300, considered toxic if swallowed. Compounds falling into Class IV have LD_50_ values from 300 to 2000, categorized as harmful if swallowed. Class V comprises compounds with LD_50_ values between 2000 and 5000, which may be harmful if swallowed. Finally, Class VI includes non-toxic compounds with LD_50_ > 5000. The results of toxicity assessment for the bioactive compounds indicated a generally low toxicity level, except for specific compounds that require close attention due to potential human hepatotoxicity (H-HT), drug-induced liver injury (DILI), hepatotoxicity, carcinogenicity, immunotoxicity, mutagenicity, and cytotoxicity when present as single compounds (Table 3 and Table 4). The average LD_50_ and toxicity class of 22 bioactive compounds ranged between Class IV and Class VI, suggesting that these compounds are predicted to be safe (Figure 4). However, it is crucial to underscore that these seven compounds (C1, C3, C8, C9, C19, C21, and C29) displayed lethal toxicity when ingested separately.

### 3.4. Potential Target Proteins and Biological Pathways

**Figure 5:**
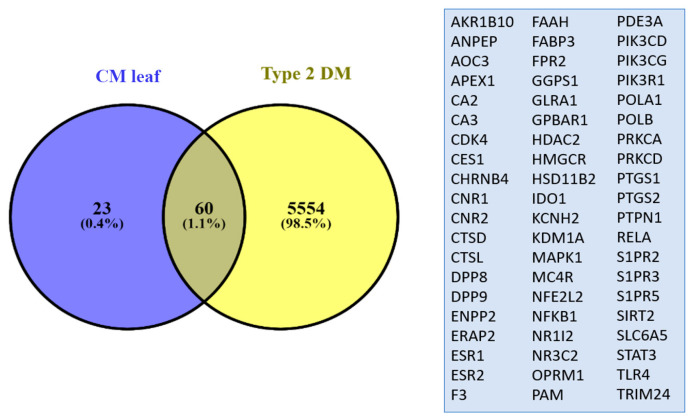
Venn diagram displaying the shared targets between the CM leaf extract and genes linked to T2DM. The CM leaf extract is suggested to have the potential to impact T2DM by modulating cytokines. This is supported by the fact that a significant number of proteins associated with the immune system are targeted by the extract, and these proteins are also associated with T2DM.

The analysis of target prediction for CM leaf extract using SEA and SuperPred revealed an intersection of 60 proteins targeted by both the CM leaf extract and T2DM. Among these 60 targeted proteins, NFE2L2, also known as Nrf2, was also identified in the previous analysis. The CM leaf extract is hypothesized to potentially influence T2DM through cytokine modulation, as a substantial number of proteins associated with the immune system are targeted by the extract and are also implicated in T2DM (Figure 5).

**Figure 6:**
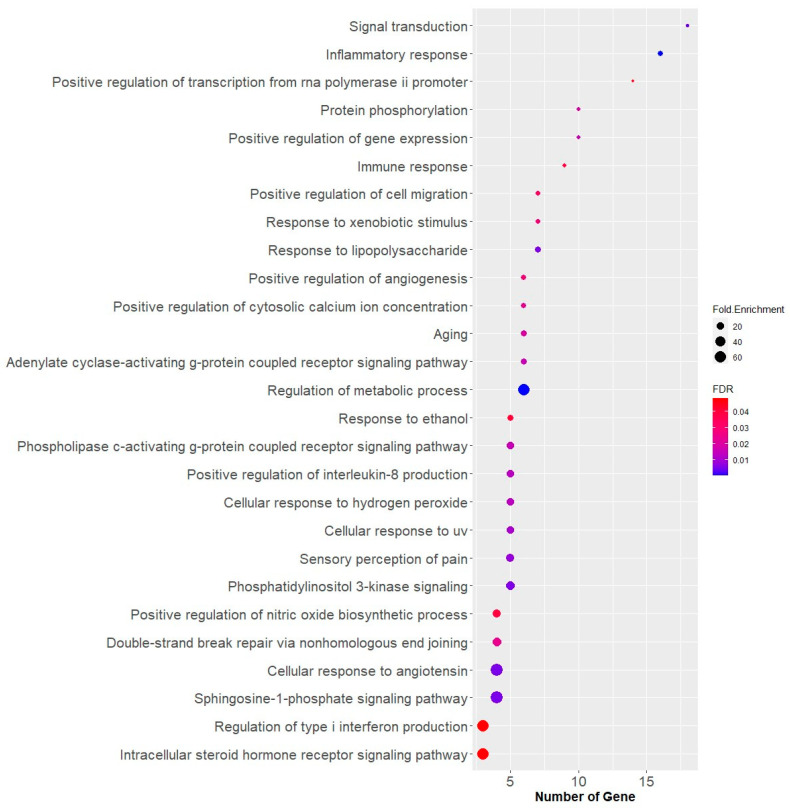
Annotation of gene ontology biological processes for CM leaf extract targets with false discovery rate (FDR) < 0.05. The enrichment score shows CM leaf extract primarily targets metabolic regulation, angiotensin response, and immune pathways. Angiotensin response is closely related to diabetes, while the extract’s main role appears to be in inflammation. Genes associated with angiotensin response, like PRKCD, RELA, NFKB1, and NFE2L2, are also involved in immune responses.

According to the gene ontology biological process analysis (Figure 6), the enrichment score indicates that the targets of CM leaf extract are primarily involved in the regulation of metabolic processes, cellular response to angiotensin, and the sphingosine-1-phosphate signaling pathway. Cellular response to angiotensin (*p*_value_ < 0.05, enrichment 65.53) is a biological process closely associated with DM. However, when considering the number of gene targets involved, the CM leaf extract appears to have a more prominent role in the inflammatory response (*p*_value_ < 0.05, enrichment 9.18). The genes involved in the cellular response to angiotensin include protein kinase C delta (PRKCD), nuclear factor-kappa B subunit p65 (RELA), nuclear factor-kappa B subunit 1 (NFKB1), and NFE2L2. Interestingly, RELA, NFKB1, and NFE2L2 are also associated with immune responses. Previous reports indicated that NFKB1 was upregulated in conditions of DM compared to control [36]. Increased NFKB1 levels have also been observed in studies comparing NFKB1 profiles between T2DM patients and normal glucose-tolerant individuals [37]. Another study also demonstrated that NFKB expression was elevated in T2DM and diabetic nephropathy samples [38]. NFKB1 was involved in the cellular response to angiotensin, where angiotensin II (ANG II) appeared to play a role in the regulation of insulin secretion by pancreatic beta cells and insulin sensitivity in peripheral tissues, two crucial factors contributing to the development of T2DM [39]. Therefore, it is suggested that NFKB1, in conjunction with its cytokine-regulating function, may play a role in insulin resistance and diabetogenesis [40].

**Figure 7:**
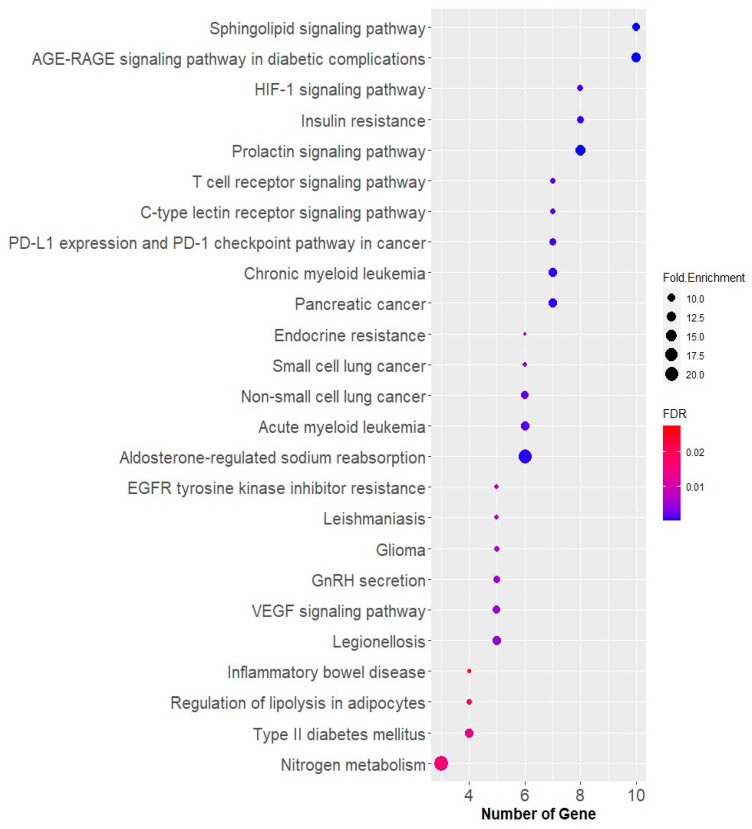
Annotation of the top 25 KEGG pathways targeted by CM leaf extract with FDR < 0.05. The most significant pathway targeted by CM leaf extract is the AGE-RAGE signaling pathway in diabetic complications. This pathway involves various proteins, including CDK4, STAT3, PRKCD, MAPK1, PIK3CD, PRKCA, PIK3R1, F3, RELA, and NFKB1. Some of these proteins are associated with inflammation, while others are involved in the PI3K receptor pathway related to T2DM. RAGE, a receptor for AGEs, plays a crucial role in this pathway, contributing to cellular stress and complications in diabetes.

Through the analysis conducted using the KEGG pathway (Figure 7), it was determined that the most significant pathway among the CM leaf extract targets was the advanced glycation end products—receptor for advanced glycation end products (AGE-RAGE) signaling pathway in diabetic complications (*p*_value_ < 0.05, enrichment 12.44). Members involved in the AGE-RAGE signaling pathway in diabetic complications include cyclin-dependent kinase 4 (CDK4), signal transducer and activator of transcription 3 (STAT3), PRKCD, mitogen-activated protein kinase 1 (MAPK1), phosphatidylinositol-4,5-bisphosphate 3-kinase catalytic subunit delta (PIK3CD), protein kinase C alpha (PRKCA), phosphatidylinositol 3-kinase regulatory subunit alpha (PIK3R1), coagulation factor III (F3), RELA, and NFKB1. NFKB1 and RELA are associated with inflammation, while the PI3K receptor pathway is involved in T2DM. RAGE is an immunoglobulin superfamily molecule, and some of its ligands accumulate in diabetic tissues. RAGE has been identified as a receptor for AGEs, such as carboxymethyl lysine (CML). AGEs primarily activate RAGE, triggering signaling mechanisms that induce cellular stress, contribute to cellular dysfunction, and damage target organs, leading to complications.

### 3.5. Network Pharmacology Insigths

**Figure 8:**
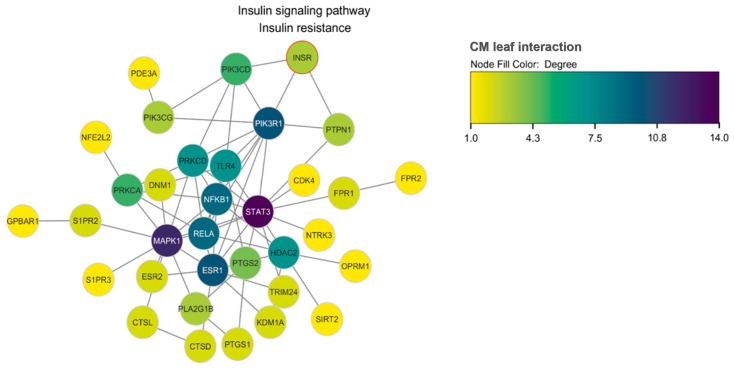
Protein–protein interaction (PPI) of CM leaf extract targets in T2DM. PIK3R1 is recognized as a prospective target of the extract, demonstrating its ability to interact with the insulin receptor (INSR). The node color intensity indicates the extent of protein interactions. This implies that the CM leaf extract is involved in pathways like PI3K/AKT signaling. Moreover, it affects inflammatory proteins, which are known to influence insulin signaling.

According to the network pharmacology analysis, PIK3R1 has been recognized as one of the potential targets of CM leaf extract that has the capability to engage with the insulin receptor (INSR). The darkness of the node color indicates the number of proteins that can interact with the analyzed protein. The findings from the network pharmacology analysis align with previous discoveries, indicating that the targeted pathways of CM leaf extract include the PI3K/AKT signaling pathway. In the protein–protein interaction (PPI) pathway, it can be observed that CM leaf extract also targets inflammatory proteins, which are known to influence insulin signaling (Figure 8).

PIK3R1 shows promise as a potential target based on the network analysis, which takes into account the degree, betweenness centrality, and closeness centrality values, combined to form an overall score. Besides its relevance to T2DM and its interaction with INSR, PIK3R1 is also involved in immune responses (Table 5). Classic centrality metrics like degree, closeness, and betweenness centrality are used to identify nodes (proteins) that play crucial roles in biological networks. Degree provides insights into how many proteins interact with a specific node. Closeness centrality estimates how quickly information flows through a node or how short the shortest paths are from node x to all other nodes. On the other hand, betweenness centrality is based on communication flow, where nodes with high betweenness centrality are significant in controlling information flow [41]. Based on classic centrality, PIK3R1 is considered a promising protein target for further investigation.

The compound profile of CM leaf extract was obtained through GC-MS analysis. This method offers significant advantages due to its comprehensive functionality. GC is capable of separating semi-volatile and volatile compounds with exceptionally high resolution; however, it falls short in terms of compound identification. In contrast, MS provides detailed structural information for most compounds, enabling precise identification, but it cannot directly separate compounds [42]. Nevertheless, GC-MS only predicts the structure, as not all molecules undergo single derivatization or readily accept derivatization [43].

α-Amyrin was detected as a bioactive component in the CM leaf extract, and it displayed the highest level of consistency when compared to other compounds. α-Amyrin is a naturally occurring triterpenoid compound present in diverse plant species. It has shown promising pharmacological potential due to its diverse biological activities. Triterpenes show significant promise in improving β-cell damage caused by diabetes by influencing various genes and proteins associated with insulin signaling, inflammation, oxidative stress, and apoptosis [44]. Numerous studies have demonstrated that these compounds possess various mechanisms with antidiabetic effects. They can inhibit enzymes related to glucose metabolism, prevent insulin resistance, and restore normal levels of plasma glucose and insulin [45].

Lipinski’s Ro5 aids in predicting the likelihood of a compound’s success or failure in terms of metabolism, depending on its resemblance to known drugs [18,46]. For a compound to adhere to Ro5, it should fulfill at least two out of five key properties, including a molecular weight less than 500 Dalton, high lipophilicity indicated by Log*P* 2-3, fewer than 10 hydrogen bond acceptors, fewer than five hydrogen bond donors, and a molar refractivity between 40 and 130. These criteria serve as the foundation for the classification and selection of potential drug candidates [46].

The discovery that RAGE interacts with non-AGE ligands, such as S100/calgranulins and high mobility group box 1 (HMGB1), suggests that RAGE is not only involved in diabetic complications but also contributes to the development of both type 1 and type 2 diabetes [47]. Among the CM leaf extract targets related to T2DM in the KEGG pathway, PRKCD, MAPK1, PIK3CD, and PIK3R1 are involved. The PI3K/AKT pathway plays a role in obesity and T2DM. Insulin-mediated PI3K/AKT signaling is blocked, leading to reduced insulin secretion and impaired β-cell function. This condition further exacerbates insulin resistance, affecting other tissues and contributing to complications [48].

The phosphoinositide 3-kinase (PI3K) pathway plays a central role in insulin metabolic action in the liver. Research using mice with specific liver deletion of the p85α PI3K regulatory subunit (L-Pik3r1KO) showed increased paradoxical insulin sensitivity in the liver and peripheral tissues. Despite reduced enzymatic PI3K activity in L-Pik3r1KO liver due to decreased levels of regulatory and catalytic subunits of PI3K, insulin-stimulated Akt activity actually increased. This elevation in Akt activity was correlated with increased levels of phosphatidylinositol (3,4,5)-trisphosphate, partially caused by reduced activity of the (3,4,5)-trisphosphate phosphatase PTEN. Thus, the regulatory p85α subunit serves as an important modulator of insulin sensitivity in vivo, not only through its influence on PI3K activation but also as a regulator of PTEN activity [49].

## 4. Conclusions

The systematic in silico evaluation of CM leaf extract as a potential treatment for T2DM has yielded significant insights. The SAR analysis identified 29 bioactive compounds with the attributes of insulin promoters, NF-E2-related factor 2 stimulants, and wound healing agents. Encouragingly, 26 of these compounds adhered to Lipinski’s Ro5 criteria, indicating their drug-likeness. ADMET analysis demonstrated that nearly all compounds displayed favorable pharmacodynamics and pharmacokinetics, with 22 bioactive compounds considered safe for use. The intersection of 60 proteins targeted by CM leaf extract and T2DM revealed the potential influence of the extract on T2DM through cytokine-related mechanisms. The functional enrichment analysis highlighted the involvement of these proteins in metabolic regulation, cellular response to angiotensin, and the sphingosine-1-phosphate signaling pathway. Network pharmacology analysis pinpointed five genes (STAT3, MAPK1, ESR1, PIK3R1, NFKB1) as potential targets of bioactive compounds, with particular emphasis on PIK3R1 due to its interaction with the insulin receptor (INSR) and its critical role in insulin signal transduction in T2DM. This study underscores the therapeutic potential of CM leaf extract in T2DM treatment by modulating essential proteins in the insulin signaling pathway. The presence of immune-response-related proteins in the target profile suggests the extract’s potential as an immunomodulator, opening avenues for future research. While the study demonstrates promise, its limitations should be acknowledged, and further investigations are required to validate its clinical applicability.

## Figures and Tables

**Table 1:** Profile of CM leaf extract’s compounds identified using GC-MS analysis.

Number	Solvent	Compounds’ Name	CID (Compound Identifier)
C1	Both	Octadecane, 6-methyl-	93065
C2	Both	Ethyl iso-allocholate	154734451
C3	Both	Octadecane, 3-ethyl-5-(2-ethylbutyl)-	292285
C4	Both	Neophytadiene	10446
C5	Both	2-Pentadecanone, 6,10,14-trimethyl-	10408
C6	Both	3,7,11,15-Tetramethyl-2-hexadecen-1-ol	5366244
C7	Both	Hexadecanoic acid, methyl ester	8181
C8	Both	(5ß)Pregnane-3,20ß-diol, 14a,18a-[4-methyl-3-oxo-(1-oxa-4-azabutane-1,4-diyl)]-, diacetate	537242
C9	Non-polar	Trichloromethane	6212
C10	Non-polar	Dithiocarbamate, S-methyl-,N-(2-methyl-3-oxobutyl)-	5363131
C11	Non-polar	2-Myristynoyl pantetheine	535560
C12	Non-polar	12-Methyl-E,E-2,13-octadecadien-1-ol	90107969
C13	Non-polar	Hexadecanoic acid, ethyl ester	12366
C14	Non-polar	Lup-20(29)-en-3-ol, acetate, (3ß)-	521518
C15	Non-polar	9,19-Cyclolanost-24-en-3-ol, acetate, (3ß)-	518616
C16	Non-polar	alpha-Amyrin	69166285
C17	Polar	3-Trifluoroacetoxypentadecane	534406
C18	Polar	Silane, trichlorodocosyl-	81761
C19	Polar	2(4H)-Benzofuranone, 5,6,7,7a-tetrahydro-4,4,7a-trimethyl-	27209
C20	Polar	2-Trifluoroacetoxypentadecane	534405
C21	Polar	2-Piperidinone, N-[4-bromo-n-butyl]-	536377
C22	Polar	9,12-Octadecadienoic acid, methyl ester, (E,E)-	5362793
C23	Polar	11-Octadecenoic acid, methyl ester	5364432
C24	Polar	Oleic acid, 3-(octadecyloxy)propyl ester	21159937
C25	Polar	Heptadecanoic acid, 16-methyl-, methyl ester	110444
C26	Polar	Phytol	5280435
C27	Polar	Stearic acid, 3-(octadecyloxy)propyl ester	551406
C28	Polar	Octadecane, 1,1’-[1,3-propanediylbis(oxy)]bis-	624534
C29	Polar	5H-Cyclopropa[3,4]benz[1,2-e]azulen-5-one, 3,9,9a-tris(acetyloxy)-3-[(acetyloxy)methyl]-2-chloro-1,1a,1b,2,3,4,4a,7a,7b,8,9,9a-dodecahydro-4a,7b-dihydroxy-1,1,6,8-tetramethyl-, [1aR-(1aa,1bß,2a,3ß,4aß,7aa,7ba,8a,9ß,9aa)]-	538181

**Table 2:** SAR profile of insulin promoters (Pa score > 0.4) from bioactive compounds of CM leaf extract.

Compounds	Solvent	Insulin Promotor Predicted Score
C1	Both	0.572
C3	Both	0.525
C7	Both	0.487
C13	Non-polar	0.553
C14	Non-polar	0.637
C16	Non-polar	0.754
C25	Polar	0.553

**Table 3:** Results of ADMET and drug-likeness analysis for potential compounds from CM leaf extract using AdmetLab 2.0.

Compounds	Pgp-Inhibitor	Pgp-Substrate	Human Intestinal Absorption	F (20% Bioavailability)	F (30% Bioavailability)	Blood–Brain Barrier	H-HT (Human Hepatotoxicity)	DILI (Drug-Induced Liver Injury)	FDAMDD (Maximum Recommended Daily Dose)	Drug-Likeness		
										Lipinski	Pfizer	GSK
C1	0.001	0	0.002	0.473	0.998	0.092	0.009	0.245	0.03	Accepted	Rejected	Rejected
C2	0.936	0.135	0.012	0.005	0.119	0.667	0.297	0.016	0.874	Accepted	Accepted	Rejected
C3	0	0.01	0.002	0.087	0.992	0.008	0.013	0.063	0.016	Accepted	Rejected	Rejected
C4	0.154	0	0.002	0.004	0.359	0.513	0.055	0.856	0.08	Accepted	Rejected	Rejected
C5	0.278	0.001	0.003	0.983	0.974	0.634	0.06	0.193	0.022	Accepted	Rejected	Rejected
C6	0.034	0.001	0.002	0.687	0.357	0.227	0.098	0.051	0.026	Accepted	Rejected	Rejected
C7	0.033	0.002	0.002	0.98	0.996	0.388	0.026	0.328	0.017	Accepted	Rejected	Rejected
C8	0.995	0	0.006	0.014	0.69	0.63	0.445	0.721	0.904	Accepted	Accepted	Rejected
C9	0.001	0.39	0.004	0	0	0.998	0.076	0.028	0.039	Accepted	Accepted	Accepted
C10	0	0.003	0.015	0.002	0.001	0.923	0.268	0.821	0.192	Accepted	Accepted	Accepted
C11	0.955	0.004	0.149	0.963	0.658	0.183	0.081	0.439	0.041	Accepted	Accepted	Rejected
C12	0	0.025	0.007	0.017	0.822	0.245	0.004	0.017	0.023	Accepted	Rejected	Rejected
C13	0.021	0.001	0.001	0.909	0.996	0.139	0.011	0.233	0.012	Accepted	Rejected	Rejected
C14	0.208	0	0.008	0.471	0.917	0.792	0.191	0.018	0.845	Accepted	Rejected	Rejected
C15	0.007	0	0.013	0.945	0.964	0.211	0.416	0.051	0.652	Accepted	Rejected	Rejected
C16	0.984	0	0.012	0.831	0.947	0.404	0.181	0.013	0.825	Accepted	Rejected	Rejected
C17	0.643	0.001	0.002	0.998	0.98	0.131	0.145	0.146	0.239	Accepted	Rejected	Rejected
C18	0	0.001	0.004	0.009	0.96	0	0.025	0.286	0.036	Accepted	Rejected	Rejected
C19	0.283	0.001	0.006	0.78	0.027	0.61	0.209	0.082	0.212	Accepted	Accepted	Accepted
C20	0.648	0	0.001	0.998	0.975	0.162	0.18	0.418	0.026	Accepted	Rejected	Rejected
C21	0.397	0	0.004	0.029	0.059	0.996	0.251	0.348	0.159	Accepted	Accepted	Accepted
C22	0.001	0.028	0.007	0.008	0.775	0.245	0.011	0.022	0.024	Accepted	Rejected	Rejected
C23	0.002	0.006	0.004	0.882	0.977	0.179	0.018	0.056	0.017	Accepted	Rejected	Rejected
C24	0	0.001	0.002	0.081	0.998	0	0.003	0.054	0.009	Rejected	Rejected	Rejected
C25	0.022	0.001	0.002	0.951	0.988	0.233	0.031	0.421	0.014	Accepted	Rejected	Rejected
C26	0.034	0.001	0.002	0.687	0.357	0.227	0.098	0.051	0.026	Accepted	Rejected	Rejected
C27	0	0	0.002	0.103	1	0	0.004	0.189	0.01	Rejected	Rejected	Rejected
C28	0	0	0.001	0.01	0.999	0	0.003	0.075	0.012	Rejected	Rejected	Rejected
C29	1	0.009	0.988	0.182	0.976	0.772	0.966	0.971	0.9	Rejected	Accepted	Rejected

Explanation: The red color indicates that the compound does not comply with Lipinski’s Rule of Five and exhibits toxicity and low bioavailability. 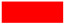: Both; 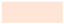: Non-polar; 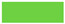: Polar.

**Table 4:** SAR profile of insulin promoters (Pa score > 0.4) from bioactive compounds of CM leaf extract.

Compounds	LD50 (mg/kg)	Toxicity Class	Average Similarity	Prediction Accuracy	Hepatotoxicity	Carcinogenicity			Immunotoxicity		Mutagenicity		Cytotoxicity	
					Prediction	Probability	Prediction	Probability	Prediction	Probability	Prediction	Probability	Prediction	Probability
C1	750	3	100	100	Inactive	0.75	Inactive	0.62	Inactive	0.96	Inactive	0.97	Inactive	0.79
C2	5000	5	91.85	72.9	Inactive	0.59	Inactive	0.77	Active	0.53	Inactive	0.73	Inactive	0.75
C3	750	3	100	100	Inactive	0.75	Inactive	0.62	Inactive	0.88	Inactive	0.97	Inactive	0.79
C4	5050	6	88.21	70.97	Inactive	0.79	Inactive	0.73	Inactive	0.99	Inactive	0.98	Inactive	0.81
C5	5000	5	100	100	Inactive	0.72	Inactive	0.75	Inactive	0.99	Inactive	0.86	Inactive	0.77
C6	5000	5	100	100	Inactive	0.79	Inactive	0.76	Inactive	0.99	Inactive	0.97	Inactive	0.85
C7	5000	5	100	100	Inactive	0.58	Inactive	0.55	Inactive	0.99	Inactive	0.98	Inactive	0.73
C8	41	2	65.82	68.07	Inactive	0.86	Active	0.62	Active	0.99	Inactive	0.68	Active	0.5
C9	36	2	100	100	Inactive	0.98	Active	0.9	Inactive	0.99	Inactive	0.94	Inactive	0.71
C10	16,000	5	62.61	68.07	Inactive	0.62	Inactive	0.58	Inactive	0.99	Inactive	0.77	Inactive	0.68
C11	10,000	6	48.98	54.26	Inactive	0.85	Inactive	0.62	Inactive	0.71	Inactive	0.89	Inactive	0.75
C12	5000	5	91.67	72.9	Inactive	0.79	Inactive	0.56	Inactive	0.94	Inactive	0.96	Inactive	0.84
C13	5000	5	100	100	Inactive	0.76	Active	0.56	Inactive	0.99	Inactive	0.99	Inactive	0.78
C14	2000	4	100	100	Inactive	0.91	Inactive	0.63	Active	0.57	Inactive	0.95	Inactive	0.97
C15	4300	5	86.84	70.97	Inactive	0.68	Inactive	0.55	Active	0.96	Inactive	0.86	Inactive	0.67
C16	1860	4	97.37	72.9	Active	0.5	Inactive	0.57	Active	0.99	Inactive	0.89	Inactive	0.96
C17	5000	5	75	69.26	Inactive	0.67	Inactive	0.54	Inactive	0.98	Inactive	0.86	Inactive	0.69
C18	2340	5	62.71	68.07	Inactive	0.85	Active	0.55	Inactive	0.98	Inactive	0.8	Inactive	0.75
C19	34	2	85.4	70.97	Inactive	0.61	Inactive	0.52	Inactive	0.91	Inactive	0.88	Inactive	0.94
C20	5000	5	75	69.26	Inactive	0.67	Inactive	0.54	Inactive	0.95	Inactive	0.86	Inactive	0.69
C21	41	2	75.7	69.26	Inactive	0.94	Inactive	0.5	Inactive	0.91	Active	0.5	Inactive	0.76
C22	20,000	6	90.37	72.9	Inactive	0.59	Inactive	0.56	Inactive	0.86	Inactive	0.98	Inactive	0.7
C23	3000	5	89.64	70.97	Inactive	0.59	Inactive	0.56	Inactive	0.96	Inactive	0.98	Inactive	0.7
C24	3520	5	85.21	70.97	Inactive	0.86	Active	0.56	Inactive	0.64	Inactive	0.81	Inactive	0.83
C25	5000	5	100	100	Inactive	0.59	Inactive	0.63	Inactive	0.99	Inactive	0.94	Inactive	0.73
C26	5000	5	100	100	Inactive	0.79	Inactive	0.76	Inactive	0.99	Inactive	0.97	Inactive	0.85
C27	1600	4	100	100	Inactive	0.86	Active	0.59	Inactive	0.88	Inactive	0.8	Inactive	0.84
C28	3000	5	84.32	70.97	Inactive	0.9	Active	0.64	Inactive	0.83	Inactive	0.86	Inactive	0.87
C29	34	2	74.51	69.26	Inactive	0.78	Active	0.5	Active	0.99	Active	0.55	Inactive	0.66

Explanation: The red color indicates that the compound does not comply with Lipinski’s Rule of Five and exhibits toxicity and low bioavailability. 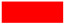: Both; 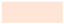: Non-polar; 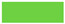: Polar.

**Table 5:** Results of the top five protein–protein interaction (PPI) network analyses conducted on the CM leaf extract.

Name	Degree	Betweenness Centrality	Closeness Centrality	Overall Score	Pathway
STAT3	14	0.369053905	0.6	14.9690539	Signal transduction, positive regulation of angiogenesis, inflammatorry response, AGE-RAGE signaling pathway in diabetic complications, insulin resistance
MAPK1	12	0.324383445	0.54098361	12.86536705	Signal transduction, AGE-RAGE signaling pathway in diabetic complications, type-2 DM, insulin signaling pathway, TNF signaling pathway
ESR1	10	0.158518286	0.54098361	10.69950189	Signal transduction, estrogen signalling pathway
PIK3R1	10	0.162430583	0.49253731	10.6549679	signal transduction, immune response, AGE-RAGE signaling pathway in diabetic complications, type-2 DM, insulin resistance, insulin signaling pathway, TNF signaling pathway
NFKB1	9	0.056809231	0.54098361	9.597792838	Immune response, AGE-RAGE signaling pathway in diabetic complications, insulin resistance, TNF signaling pathway

## Data Availability

The data that support the findings of this study are available from the corresponding author upon reasonable request.
